# Gait instability, ophthalmoplegia, and chorea with orofacial dyskinesia in a man with anti-Ri antibodies: a case report

**DOI:** 10.3389/fneur.2024.1359781

**Published:** 2024-03-20

**Authors:** Mukuto Shioda, Hiroaki Fujita, Hiroki Onuma, Hirotaka Sakuramoto, Mai Hamaguchi, Keisuke Suzuki

**Affiliations:** Department of Neurology, Dokkyo Medical University, Mibu, Tochigi, Japan

**Keywords:** paraneoplastic neurological syndrome, anti-Ri antibody, chorea with orofacial dyskinesia, respiratory failure, case report

## Abstract

A 79-year-old man was admitted for 2 weeks of dizziness, followed by diplopia, involuntary movement and progressive gait disturbances. Neurologic examination revealed horizontal and vertical gaze paresis, bilateral choreiform movement with orofacial dyskinesia, and limb/truncal ataxia. MRI revealed fluid-attenuated inversion recovery image-hyperintense signal abnormalities in the dorsal midbrain, pontine and medulla. Within another few days, the patient developed type II acute respiratory failure requiring artificial invasive ventilation. Because autoimmune encephalitis was suspected, he received intravenous immunoglobulin therapy followed by intravenous methylprednisolone, but only his ophthalmoplegia improved minimally. Serological tests were positive for anti-Ri onconeural antibodies. CT-guided mediastinal lymph node biopsy was performed and revealed small cell lung carcinoma. We report the rare manifestation of anti-Ri antibody-associated paraneoplastic neurological syndrome (PNS), and this case can alert us to the importance of respiratory management in this diverse neurologic disease. Furthermore, PNSs positive for anti-Ri antibodies should be added to the list of differential diagnoses of chorea with orofacial dyskinesia.

## Introduction

Paraneoplastic neurological syndrome (PNS) can be caused by altered immune reactions mediated by distant tumors. These reactions can be described as conditions that result from the indirect effects of cancer secondary to antigen–antibody interactions incited by the underlying malignancy. Onconeural antibodies are antibodies against intracellular antigens and are considered high-specific markers of a paraneoplastic etiology. Anti-Ri antibody-associated PNSs are known to exhibit characteristic symptoms such as opsoclonus-myoclonus syndrome (OMS) and ataxia, but heterogeneous clinical manifestations have been reported (1). This rare PNS is more common in women with breast cancer and in men with bladder cancer, lung cancer or seminoma (2). We herein report a patient with anti-Ri antibody-positive PNS who presented with characteristic clinical manifestations followed by acute respiratory failure.

## Case description

A 79-year-old man presented with a two-week history of dizziness, which was followed by diplopia, involuntary hyperkinetic movements of both the arms and face, and an unsteady gait. His medical history included type II diabetes and hypertension, which were well controlled with oral medications. He did not take any psychiatric agents. He repeatedly fell and felt dyspnea upon exertion. On admission, he was afebrile and had an oxygen saturation of 98% with 3 L of inhaled oxygen. He remained oriented but was not clearly conscious and seemed agitated. Neurologic examination revealed horizontal and vertical gaze palsy in both eyes without opsoclonus. Normal direct and indirect pupillary responses were observed. There was no facial muscle weakness or sensory deficits. Prominent bilateral choreiform movements were observed (see Supplementary Video S1), and these movements appeared during the day and disappeared during sleep. Repeated involuntary movements of his face, such as frowning and sticking out his tongue, were observed (see Supplementary Video S2), resulting in hyperkinetic dysarthria. No signs of meningeal irritation were noted. He had full motor power and no sensory deficits. His reflexes were decreased in the lower extremities. He had bilateral ataxia on finger-to-nose testing, dysdiadochokinesia, and heel-to-shin dysmetria. Due to severe truncal ataxia, he had difficulty holding an end-sitting or standing position.

**Figure 1:**
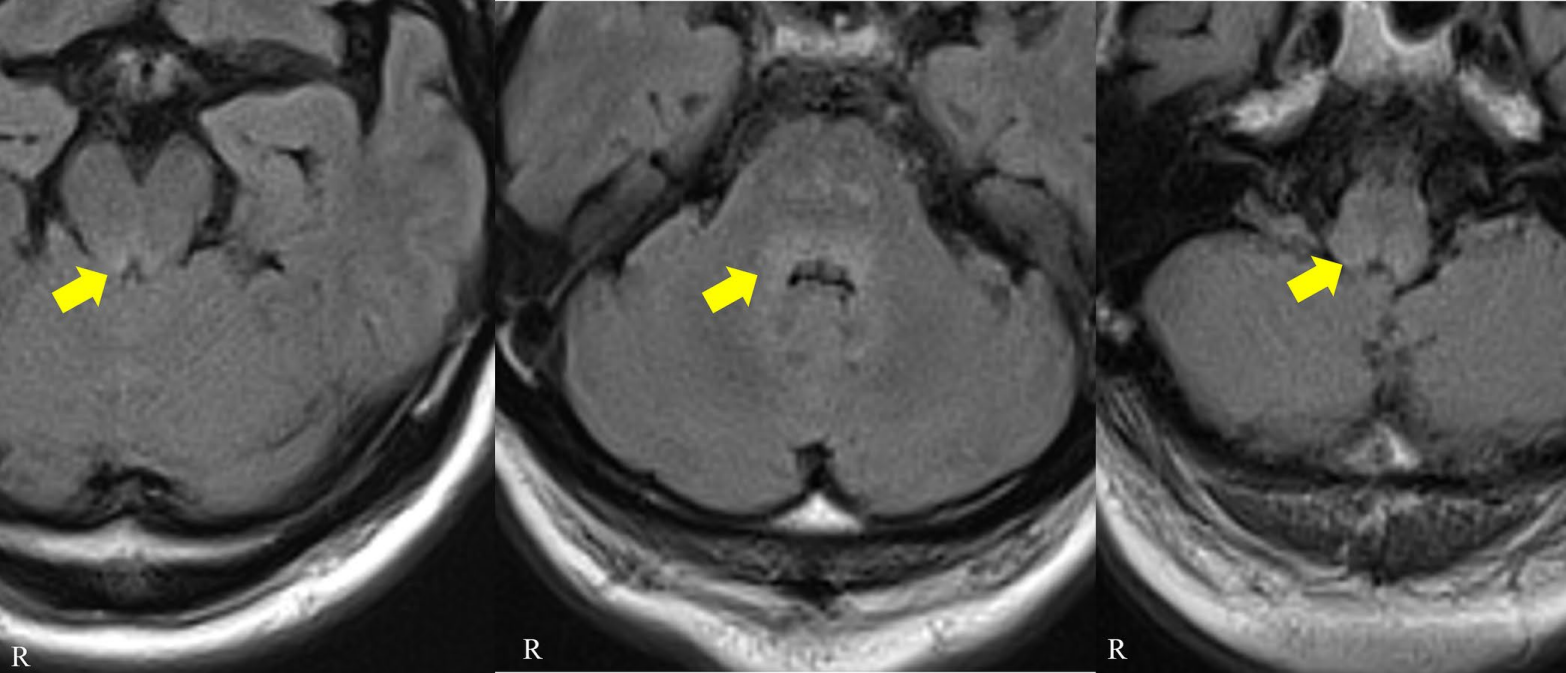
Patient brain MRI. Axial fluid-attenuated inversion recovery images showing hyperintense signals in the dorsal midbrain, pons and medulla (arrows).

The blood cell counts were normal with normal erythrocyte morphology on the blood smear. Basic serum biochemistry tests showed only nonspecific findings, with elevated C-reactive protein and creatine kinase and reduced sodium results. The aquaporin-4 antibody, antinuclear antibody, interferon-gamma release assay, and angiotensin converting enzyme results were negative or within the normal range. Anti-thyroid peroxidase (24 IU/mL, normal range < 16) and anti-thyroglobulin antibodies (51.2 IU/mL, normal range < 28) were positive, but the patient had normal thyroid hormone levels. His glutamic acid decarboxylase antibody level was mildly elevated (35 U/L, normal range < 5). He had a normal glucose level with a glycated hemoglobin level of 6.9% (National Glycohemoglobin Standardization Program). A screening test for tumor markers revealed elevated pro-gastrin-releasing peptide (121 pg./mL, normal range < 81), squamous cell carcinoma antigen (3.3 ng/mL, normal range < 1.5), and neuron-specific enolase (25.7 ng/mL, normal range < 16.3%). A cerebrospinal fluid examination revealed mild lymphocyte pleocytosis (24 cells/mm^3^, normal range < 5), mild elevation in protein (53.6 mg/dL) with a high IgG index (0.81), and negativity for cytology and viral markers. A brain MRI revealed fluid-attenuated inversion recovery image-hyperintense lesions in the dorsal medulla, pons, and midbrain with no associated enhancement or restricted diffusion (Figure 1). There was no abnormal signal or atrophy in the basal ganglia. An electroencephalogram did not reveal epileptiform discharges.

**Figure 2:**
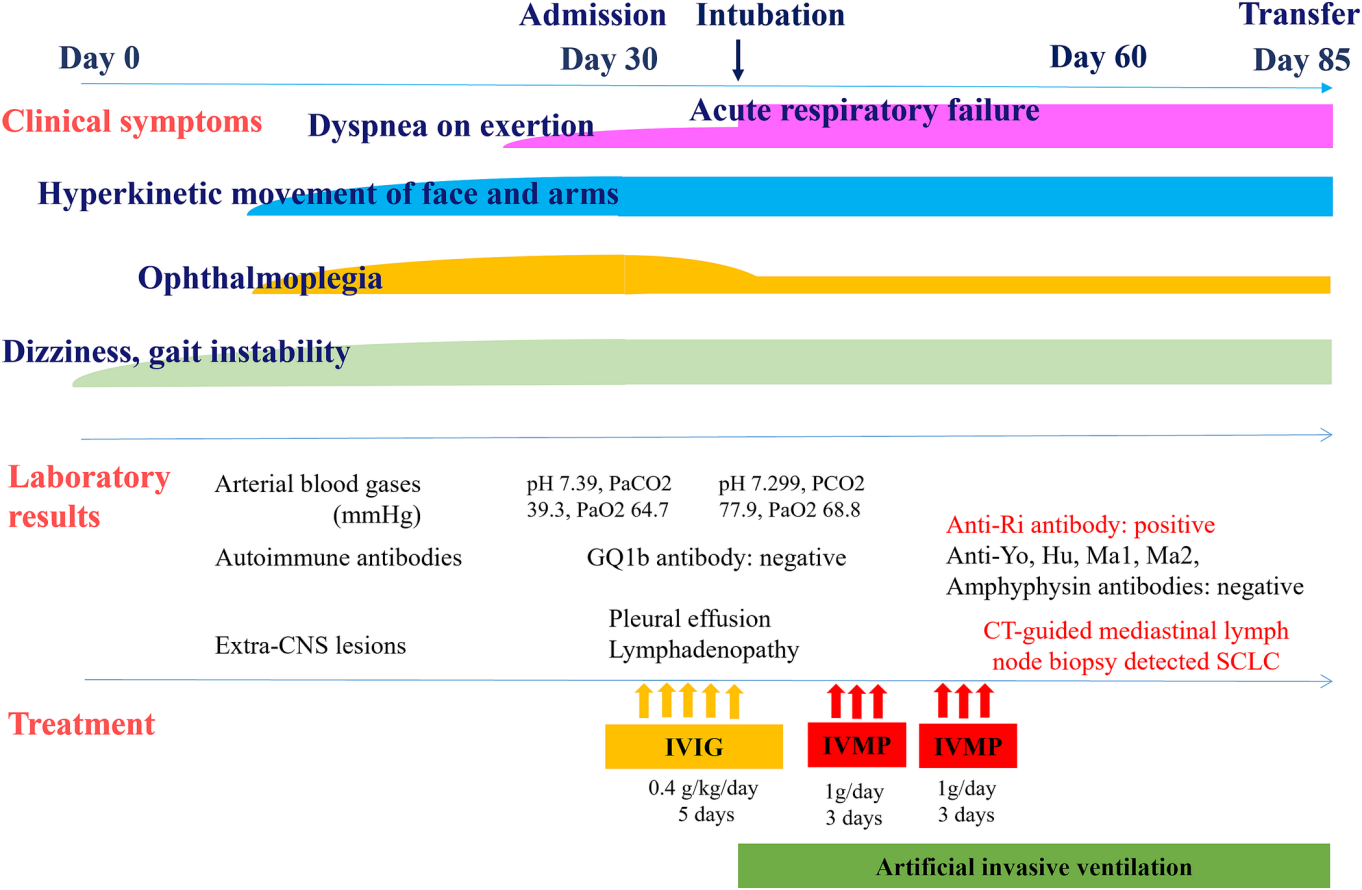
Clinical course and treatment of the patient.

Figure 2 shows the clinical course and treatment of the patient. On day 3 of admission, he further deteriorated with hypercapnic hypoxic respiratory failure (pH, 7.299; PaCO_2_, 77.9 mmHg; PaO_2_, 68.8 mmHg) requiring artificial invasive ventilation. Because autoimmune encephalitis, such as Bickerstaff brainstem encephalitis, was suspected, he received intravenous immunoglobulin (0.4 g/kg/day for 5 days) followed by two courses of intravenous methylprednisolone each for three days, with partial recovery of the ophthalmoplegia; however, the treatment had no effect on his hyperkinetic movement or respiratory failure. Subsequently, he was found to be negative for the anti-GQ1b antibody. Serological tests were positive for anti-Ri onconeural antibodies but negative for anti-Yo, anti-Hu, anti-Ma1, anti-Ma2, anti-amphiphysin, and anti-CV2/collapsin response mediator protein 5 (CRMP5). The antibodies against N-methyl-D-aspartate receptor (NMDAR), leucine-rich glioma-inactivated protein 1 (LGI1), contactin-associated protein 2, α-amino-3-hydroxy-5-methyl-4-isoxazolepropionic acid receptor, γ-amino butyric acid B receptor, dipeptidyl aminopeptidase-like protein 6 and myelin oligodendrocyte glycoprotein in the serum and cerebrospinal fluid were all negative.

Chest contrast-enhanced computed tomography (CT) revealed no mass in the pulmonary field, but pleural effusion and mediastinal lymphadenopathy, including in the para-aortic lymph nodes, were noted (Supplementary Figure S1). CT-guided mediastinal lymph node biopsy was performed and revealed small cell lung carcinoma (Supplementary Figure S2). This patient was diagnosed with anti-Ri antibody-associated paraneoplastic brainstem encephalitis with small cell lung cancer. He and his family refused any treatment for his lung cancer, and he was then transferred to another facility.

## Discussion

The present case showed a characterized and rare presentation of PNS with positivity for anti-Ri antibodies, such as ophthalmoplegia, chorea with orofacial dyskinesia and gait disturbance, and these symptoms were followed by acute respiratory failure. The anti-Ri antibody is a marker of CD8^+^ T-cell-mediated PNS and is strongly associated with cancer. The clinical manifestations result from a central nervous system injury to neuroanatomic regions that express neuro-oncological ventral antigen (NOVA)-1 and NOVA-2, which are RNA-binding proteins that regulate neuronal pre-mRNAs that are mainly expressed in the dorsal brainstem and cerebellum (3). Autopsies of anti-Ri PNS patients revealed neuronal cell loss with astrogliosis in the brainstem and Purkinje cell loss with Bergmann gliosis in the cerebellum. Immunohistochemistry has shown a predominance of B cells and CD4^+^ T cells in perivascular spaces and cytotoxic CD8^+^ T cells in parenchymal infiltrates (4). The production of anti-Ri antibodies results from an immune-mediated response against processed onconeural polypeptides presented to CD4+ T helper cells on major histocompatibility complex (MHC) class I. Among parenchymal inflammatory cells, Ri-specific cytotoxic CD8^+^ T lymphocytes conceivably target neurons that express Nova-derived peptides in the context of upregulated MHC class 1 (4).

Although OMS was previously thought to be the classical manifestation of anti-Ri PNS (5), Simard et al. (2) claimed that only 25% of patients in their French cohort presented with OMS. Recently, the clinical phenotype of PNSs positive for the anti-Ri antibody has been considered more divergent. Basically, patients with anti-Ri antibodies present with neurologic syndromes involving the brainstem or cerebellum, such as limb and truncal ataxia resulting in gait disturbances and ophthalmoplegia with and without OMS (1). Atypical symptoms and signs, such as isolated confusion, syndrome of inappropriate antidiuretic hormone secretion, and dysautonomia with central hypoventilation, were also detected in a minority of the patients (2). In a retrospective case series of 28 patients with anti-Ri antibodies (1), the most common initial symptom was gait instability (86%). Horizontal gaze palsy (21%) and jaw opening dystonia (14%) were also common accompanying symptoms. Similarly, a retrospective French cohort of 36 patients with anti-Ri (2) showed four main symptoms at disease onset, and these included cerebellar syndrome (39%), which presented with gait instability/ataxia and an action tremor; an isolated tremor (25%); oculomotor disturbances (17%); and other symptoms (19%). Our patient also initially suffered from diplopia and gait disturbances because of truncal ataxia, and these symptoms are considered common clinical manifestations of anti-Ri PNS.

Chorea with orofacial dyskinesia was the most striking manifestation in our patient. The most common antibody identified in paraneoplastic chorea is CRMP-5/CV2, followed by anti-Hu and NMDAR antibodies (6). Patients with paraneoplastic chorea can be normal on brain MRI or can exhibit extensive T2 hyperintense signals in the bilateral basal ganglia (7). To our knowledge, only one case of anti-Ri antibody-positive paraneoplastic chorea has been reported in the literature (8). That report presented the disease course of a 60-year-old woman who initially developed acute schizoaffective psychosis. Two weeks later, her choreiform movements gradually occurred predominantly on her left side. After two months, she was diagnosed with ductal carcinoma *in situ* in the left breast. She underwent mastectomy, which was followed by the administration of chemotherapy and anastrozole. Although her breast cancer was treated effectively, her choreiform movements were persistent and generalized. Positive results for anti-Hu and anti-Ri antibodies were obtained. Brain MRI revealed bilateral atrophy in the caudate, putamen, parahippocampal gyrus and hippocampus. The patient received amantadine sulfate and intravenous methylprednisolone, but neither had any effect on her chorea. In the literature, there have been no other case reports of paraneoplastic chorea with positivity for anti-Ri antibodies; there was only one of 14 patients with paraneoplastic chorea in the 2013 review (9) and one of 28 patients with anti-Ri antibodies in the 2003 review (1). In all three of these patients, anti-Hu antibodies were simultaneously positive (Table 1). The detected tumors were SCLC (one case was not described in detail) and breast cancer. In the review by O’Toole, a favorable response to chemotherapy and immunotherapy was obtained in one of the patients, with regard to the patient’s chorea, but the patient in the case reported by Martinková and our patient did not show any improvement in their chorea with treatment. Furthermore, jaw dystonia is a common involuntary movement of the face in anti-Ri PNS patients (10). Orofacial dyskinesia, including grimacing, forceful jaw opening and closing, and masticatory-like movements, is often observed in patients with paraneoplastic chorea with positivity for anti-CRMP5/CV2, anti-Hu and anti-NMDAR antibodies (5) but has also been reported in patients with anti-LGI 1 (11) or anti-voltage-gated potassium channel complex antibodies (12). To our knowledge, this is the first case of anti-Ri PNS without concomitant anti-CRMP5/CV2 or anti-Hu antibodies in which choreiform movements with orofacial dyskinesia was a striking clinical feature.

**Table 1:** Patients positive for anti-Ri antibody presenting as paraneoplastic chorea

Author	Patients (age, y/sex)	Tumor	Other sings	Chorea characteristic	Immunotherapy/chorea outcome	Oncologic therapy/chorea outcome	Brain MRI	Concomitant antibodies
Pittock et al. 2003 (in review) (1)	50, female	Chest lesion (details not described)	Cerebellar symptoms, peripheral neuropathy, dysphagia	Not described	Not described	Not described	Not described	Anti-Hu
O’Toole et al. 2013 (in review) (9)	63, male	SCLC	Sensory ganglionopathy, myelopathy	Initially, focally occurring in the limbs, then generalized	PLEX, CTX, IVMP/Improved from bedbound to ability to mobilize with a walker	Chemotherapy/gradual improvement over time	Not described	Anti-Hu, CRMP-5/CV2
Martinková et al. 2009 (8)	60, female	Breast cancer (ductal carcinoma in situ)	Schizoaffective psychosis	Initially, hemichorea, then generalized	Amantadine sulfate, IVMP/no improvement	Mastectomy, chemotherapy, anastrozole/no improvement	Atrophy in the caudate, putamen, para-hippocampal gyrus, hippocampus	Anti-Hu
Our case	79, male	SCLC	Gait disturbance, ophthalmoplegia, orofacial dyskinesia, respiratory failure	Bilateral chorea with orofacial dyskinesia	IVIg, IVMP/no improvement	Not applicable	FLIAR-hyperintensity in the dorsal brainstem without basal ganglia involvement	GAD 65 (low titer)

CRMP-5, collapsin response-mediator protein 5; SCLC, small cell lung carcinoma; IVIg, intravenous immunoglobulins; IVMP, intravenous methylprednisolone; PLEX 5, plasma exchange; CTX, cyclophosphamide; FLAIR, fluid-attenuated inversion recovery; GAD, glutamic acid decarboxylase.

Central hypoventilation, especially during sleep (known as Ondine syndrome), may occur in a minority of Ri-PNS patients (2, 13). The condition was considered to affect the dorsal pontine, causing horizontal gaze palsy, and the pathology then extended downward to the medulla oblongata, causing Ondine’s curse. Acquired central hypoventilation may result from pathologic involvement of the brainstem respiratory nuclei, as has been observed in other autoimmune diseases, such as anti-Hu brainstem syndrome and anti-NMDAR encephalitis (14, 15). Tay et al. (16) reported a case similar to our patient who presented with confusion, horizontal gaze palsy, gait disturbance, hemiataxia and SIADH, subsequent bulbar involvement and type II respiratory failure. Positive results for anti-Ri antibody and negative results for anti-Yo, anti-Hu and anti-Ma antibodies were obtained. Postmortem findings revealed CD8^+^ T-cell-centered lymphocytic infiltration, particularly in the pons, medulla and circulatory and respiratory centers. Vigliani et al. (17) reported a man with brainstem encephalitis who was positive for both anti-Hu and anti-Ri antibodies and who subsequently developed acute respiratory failure. The postmortem findings included diffuse neuronal loss and reactive gliosis throughout the whole brain stem. Velazquez et al. (18) reported a 64-year-old man who presented progressive muscle weakness, hypersomnia, tongue myoclonus, horizontal gaze palsy, ptosis and ventilator-dependent respiratory failure. He was positive for anti-Ri antibodies, and a peripancreatic lymph node biopsy showed poorly differentiated carcinoma from a pancreaticobiliary or pulmonary origin. His brain MRI was normal, and his brain pathology was not described. Stewart et al. (19) reported a rare case of nasopharyngeal carcinoma that was positive for anti-Ri antibodies and presented with opsoclonus, facial and limb myoclonus, truncal ataxia and type II respiratory failure. Brain MRI revealed abnormal signals in the left posterior medulla oblongata that extended into the left cerebellar peduncle. In our case, horizontal gaze palsy indicated lesions in the dorsal pontine, and vertical gaze palsy and hypoventilation suggested extension of the lesion to the mesencephalon and medulla, which was supported by the MRI findings. Although no other reports of hypoventilation in anti-Ri-PNS patients were found in our search, hypoventilation may cause sudden death, indicating the importance of cardiopulmonary monitoring in patients with anti-Ri-PNS.

## Conclusion

We report a case of anti-Ri antibody-associated paraneoplastic brainstem encephalitis with small cell lung cancer that presented as gait instability, ophthalmoplegia, cerebellar symptoms, chorea with orofacial dyskinesia and acute hypoventilation. This report expands the body of knowledge on the clinical manifestations associated with anti-Ri antibodies and alerts us to the importance of respiratory management in these patients. Furthermore, PNSs positive for anti-Ri antibodies should be added to the list of differential diagnoses for patients with choreiform movement and orofacial dyskinesia.

## Data availability statement

The original contributions presented in the study are included in the article/Supplementary material, further inquiries can be directed to the corresponding author.

## Ethics statement

The studies involving humans were approved by the Institutional Ethical Committee of Dokkyo Medical University. The studies were conducted in accordance with the local legislation and institutional requirements. The patient provided their written informed consent to participate in this study. Written informed consent was obtained from the individual for the publication of any potentially identifiable images or data included in this article.

## Author contributions

MS: Writing – review & editing, Writing – original draft, Investigation, Data curation, Conceptualization. HF: Writing – review & editing, Writing – original draft, Visualization, Supervision, Project administration, Methodology, Investigation, Formal analysis, Conceptualization. HO: Writing – review & editing, Writing – original draft, Validation, Supervision, Conceptualization. HS: Writing – review & editing, Writing – original draft, Supervision, Project administration, Methodology, Conceptualization. MH: Writing – review & editing, Writing – original draft, Visualization, Validation, Project administration. KS: Writing – review & editing, Writing – original draft, Validation, Supervision, Project administration.

## Supplementary material

SUPPLEMENTARY FIGURE S1 Chest contrast-enhanced computed tomography Pleural effusion and para-aortic lymphadenopathy were noted (arrow).

SUPPLEMENTARY FIGURE S2 The cytological finding of the lymph node biopsy sample revealed small cell lung carcinoma with small-sized neoplastic cells, high nuclear/cytoplasmic ratio, scant cytoplasm, fine granular chromatin and molding (Papanicolaou stain; original magnification, x1000).
