# Supplementary figures and images for: Gait instability, ophthalmoplegia, and chorea with orofacial dyskinesia in a man with anti-Ri antibodies: a case report
Source: Front Neurol. 2024 Mar 20;15:1359781. doi: 10.3389/fneur.2024.1359781 (PMC10987690; doi:10.3389/fneur.2024.1359781)

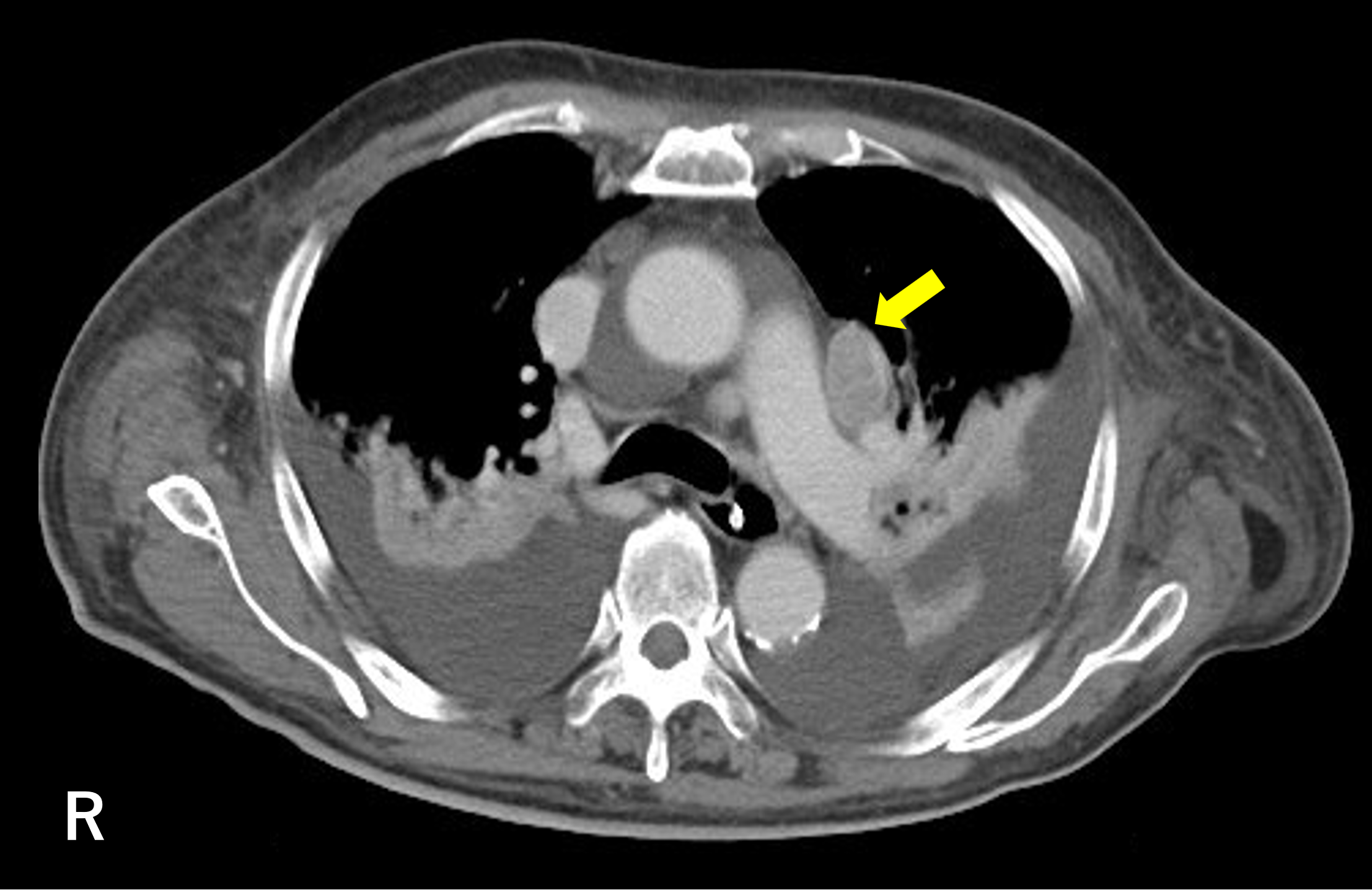

Supplement: SUPPLEMENTARY FIGURE S1 — Chest contrast-enhanced computed tomography Pleural effusion and para-aortic lymphadenopathy were noted (arrow). [file Image_1.tif]

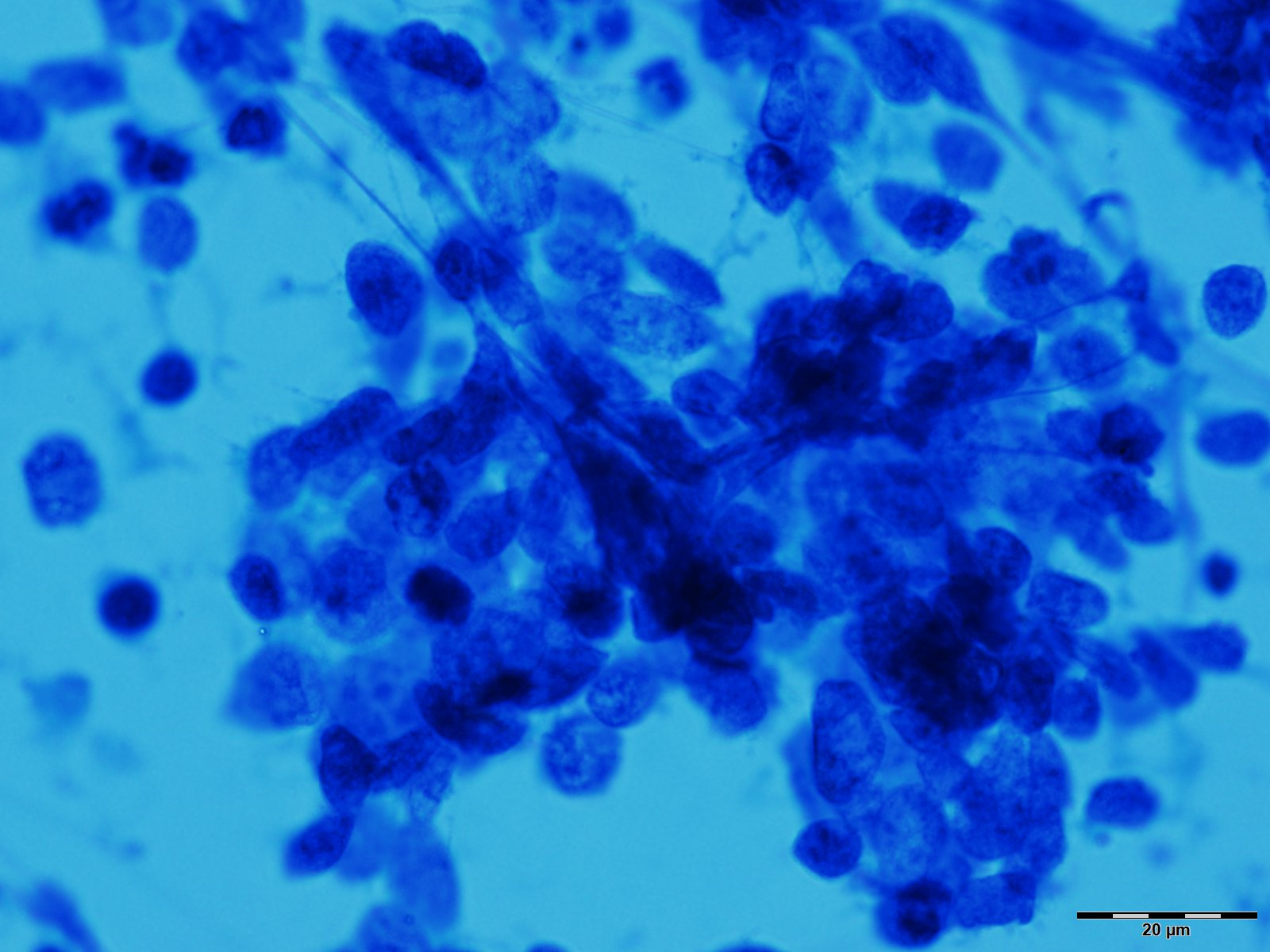

Supplement: SUPPLEMENTARY FIGURE S2 — The cytological finding of the lymph node biopsy sample revealed small cell lung carcinoma with small-sized neoplastic cells, high nuclear/cytoplasmic ratio, scant cytoplasm, fine granular chromatin and molding (Papanicolaou stain; original magnification, x1000). [file Image_2.tif]
